# Lysosomal sequestration of hydrophobic weak base chemotherapeutics triggers lysosomal biogenesis and lysosome-dependent cancer multidrug resistance

**DOI:** 10.18632/oncotarget.2732

**Published:** 2014-12-31

**Authors:** Benny Zhitomirsky, Yehuda G. Assaraf

**Affiliations:** ^1^ The Fred Wyszkowski Cancer Research Laboratory, Dept. of Biology, Technion-Israel Institute of Technology, Haifa 32000, Israel

**Keywords:** Chemotherapeutics, Multidrug resistance, Lysosomes, Drug sequestration, Lysosomal biogenesis

## Abstract

Multidrug resistance (MDR) is a primary hindrance to curative cancer chemotherapy. In this respect, lysosomes were suggested to play a role in intrinsic MDR by sequestering protonated hydrophobic weak base chemotherapeutics away from their intracellular target sites. Here we show that intrinsic resistance to sunitinib, a hydrophobic weak base tyrosine kinase inhibitor known to accumulate in lysosomes, tightly correlates with the number of lysosomes accumulating high levels of sunitinib in multiple human carcinoma cells. Furthermore, exposure of cancer cells to hydrophobic weak base drugs leads to a marked increase in the number of lysosomes per cell. Non-cytotoxic, nanomolar concentrations, of the hydrophobic weak base chemotherapeutics doxorubicin and mitoxantrone triggered rapid lysosomal biogenesis that was associated with nuclear translocation of TFEB, the dominant transcription factor regulating lysosomal biogenesis. This resulted in increased lysosomal gene expression and lysosomal enzyme activity. Thus, treatment of cancer cells with hydrophobic weak base chemotherapeutics and their consequent sequestration in lysosomes triggers lysosomal biogenesis, thereby further enhancing lysosomal drug entrapment and MDR. The current study provides the first evidence that drug-induced TFEB-associated lysosomal biogenesis is an emerging determinant of MDR and suggests that circumvention of lysosomal drug sequestration is a novel strategy to overcome this chemoresistance.

## INTRODUCTION

Chemotherapy remains the primary treatment modality of various human malignancies. However, multidrug resistance (MDR), a phenomenon in which tumor cells display resistance to multiple structurally and functionally unrelated anticancer drugs, is a primary hindrance to curative cancer therapy [1–3]. Numerous mechanisms of MDR have been described including decreased drug uptake, increased drug efflux via overexpression of MDR efflux transporters of the ATP-binding cassette (ABC) superfamily, quantitative and qualitative alterations in intracellular drug target proteins, as well as evasion of apoptosis [1, 3–8]. Since MDR is believed to be one of the leading causes of the limited success of multiple chemotherapeutic regimens, there is a burning need to decipher the molecular mechanisms underlying inherent and acquired drug resistance. This should pave the way towards the development of novel strategies to overcome various mechanisms of MDR [9, 10].

Lysosomes are acidic intracellular organelles containing various acidic hydrolases that play a key physiologic role in the breakdown of macromolecules including nucleic acids, proteins, lipids and polysaccharides. Moreover, lysosomes have been shown to take active part in various cellular processes including recycling of defective organelles, engulfment of bacteria and viruses, exocytosis, apoptosis and autophagy [11–13]. Recently, lysosomal biogenesis was found to be triggered by the translocation of transcription factor EB (TFEB) from the cytoplasm to the nucleus [14]. TFEB is a master regulator that activates the transcription of genes in the Coordinated Lysosomal Expression and Regulation (CLEAR) network responsible for the biogenesis and function of lysosomes [12, 15, 16]. TFEB-mediated lysosomal biogenesis was shown to be induced by different stimuli such as aberrant lysosomal storage, cell starvation and pharmacologic inhibition of the key growth regulator, mTOR complex 1 (mTORC1) [14]. The latter is a kinase that was shown to inhibit TFEB activity by phosphorylating it and preventing its translocation from the cytoplasm to the nucleus. Specifically, TFEB is phosphorylated at Ser211 in an mTORC1-dependent manner; this TFEB phosphorylation promotes its association with members of the YWHA (14-3-3) family of proteins resulting in the retention of TFEB in the cytosol in a transcriptionally inactive form [17].

Lysosomes have been shown to sequester lipophilic amine drugs through a non-enzymatic and non-transporter mediated process known as lysosomal trapping or lysosomal sequestration [18–21]. Lipophilic amine drugs with weak base properties readily diffuse across cell membranes at physiologic pH via passive diffusion. However, upon entry into acidic lysosomes and late endosomes, they undergo protonation and are hence entrapped in lysosomes in their cationic state [22]. In this respect, we as well as others have previously shown that certain hydrophobic weak base chemotherapeutics such as the anthracyclines doxorubicin, daunorubicin and mitoxantrone, imidazoacridinones, as well as the multitargeted receptor tyrosine kinase inhibitor sunitinib, highly accumulate in lysosomes [20, 23–28] (Table 1), thereby being sequestered away from their intracellular target sites. Indeed, it has been suggested that lysosomal drug sequestration may abolish the drug-induced cytotoxic effect, due to markedly decreased drug concentrations at the target site [18, 29]. In this respect, it has been previously demonstrated that the extent of lysosomal drug sequestration depends on the pH gradient between the acidic luminal pH of the lysosome and the cytoplasm [30]. Hence, lysosomal drug accumulation can be reversed by cell treatment with lysosome alkalinizing agents such as bafilomycin A1, a vesicular H^+^-ATPase inhibitor [25, 31]. These findings suggest that well-tolerated lysosome alkalinization agents may be used to increase cytotoxic drug efficacy by abolishing lysosomal drug sequestration. Moreover, it has been shown that differences in the lysosome-cytosol pH gradient between MDR cancer cells and their parental drug sensitive cell lines have a major impact on differential lysosomal drug accumulation in these cells, and thus on intracellular drug distribution and pharmacologic activity [30]. Interestingly, some recent evidence suggests that certain MDR transporters of the ABC superfamily such as ABCB1 (P-gp) are overexpressed and targeted to the lysosomal membrane, thereby further actively enhancing ATP-driven intralysosomal drug sequestration [32, 33].

**Table 1:** Characteristics relating to lysosomal sequestration of various drugs The calculated log P and pKa values as predicted by ChemAxon software were obtained from the drugbank database.

Therapeutic Indication	Drug	Calculated LogP	Calculated pKa (strongest basic)	Lysosomal sequestration	Reference^a^
Anticancer	Daunorubicin	1.73	8.94	+	[48, 49]
	Doxorubicin	0.92	8.94	+	[24, 28]
	Gefitinib	3.75	6.85	+	[20]
	Lapatinib	4.64	7.20	+	[20]
	Mitoxantrone	1.19	9.08	+	[26, 27]
	Pyrimethamine	2.75	7.77	+	[29]
	Sunitinib	2.93	9.04	+	[23]
	Vincristine	3.13	8.66	+	[41]
	5-Fluorouracil	−0.66	−8.00	−	
	Pemetrexed	0.73	0.96	−	
Antidepressant	Desipramine	3.90	10.02	+	[20]
	Fluoxetine	4.17	9.80	+	[20]
	Imipramine	4.28	9.20	+	[20]
	Paroxetine	3.15	9.77	+	[20]
Antimalarial	Chloroquine	3.93	10.32	+	[20]
	Mefloquine	4.11	9.46	+	[44]
	Quinacrine	5.15	10.33	+	[50]
β-adrenergic blocker	Propranolol	2.58	9.67	+	[20]

a The references in the right most column refer to lysosomal sequestration of each drug.

Collectively, these findings suggest that lysosomes may be an important determinant of intrinsic chemoresistance, and that a better understanding of the mechanisms underlying lysosome-mediated drug resistance may lead to the discovery of novel treatment strategies that could readily overcome this type of MDR [9, 10]. As a step towards this end, we here investigated the impact of lysosomal accumulation of hydrophobic weak base anticancer drugs on the onset of lysosomal biogenesis, and on the increase in lysosome number in cancer cells. We show that the number of lysosomes which accumulated high levels of sunitinib, remarkably correlates with the intrinsic resistance to this anti-tumor agent in multiple human carcinoma cells of distinct tissue lineage. We further demonstrate that hydrophobic weak base drugs induce a marked increase in lysosomal biogenesis that is mediated by induction of the CLEAR pathway regulated by nuclear translocation of TFEB. Hence, drug-induced lysosomal biogenesis and lysosomal drug sequestration is an emerging determinant of MDR, and abrogation of lysosomal drug sequestration may overcome this mode of chemoresistance.

## RESULTS

### Lysosomal drug accumulation protects nuclei from cytotoxic drugs

**Figure 1:**
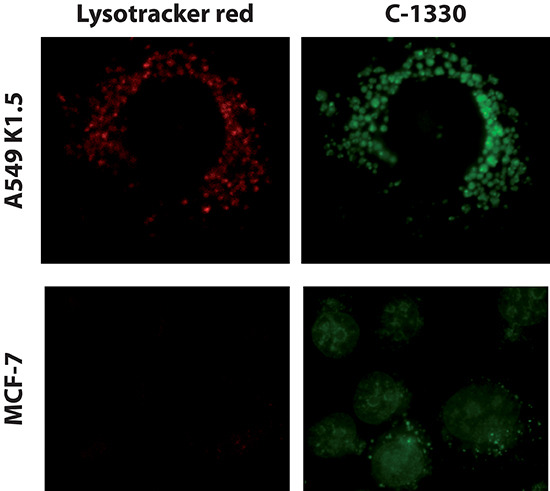
Lysosome-mediated protection of nuclear DNA from hydrophobic weak base cytotoxic agents MCF-7 breast cancer cells and A549/K1.5 non-small cell lung cancer cells were incubated for 1 hr with 100 nM LysoTracker red (red fluorescence) and 10 μM of the imidazoacridinone C-1330 (green fluorescence). Fluorescence microscopy analysis was performed using a Zeiss inverted Cell-Observer Axiovert 200 microscope (Carl Zeiss, Oberkochen, Germany).

Here we postulated that lysosomal sequestration of hydrophobic weak base anticancer drugs prevents their accessibility to their intracellular target sites, hence abolishing their pharmacologic activity. In this respect, we hypothesized that cells with an increased lysosome number have an enhanced lysosomal sequestration capacity of hydrophobic weak base cytotoxic drugs. This should result in a markedly decreased drug concentration at the drug target site and consequently increased drug resistance. To test this hypothesis, MCF-7 cells which we found to contain a low number of lysosomes, as well as A549/K1.5 MDR non-small cell lung cancer cells, known to harbor high levels of lysosomes [25], were exposed for 1 hr to the imidazoacridinone C-1330 (10 μM) along with the viable lysosome fluorescent marker LysoTracker red (100 nM), followed by fluorescence microscopy. C-1330 is a naturally fluorescent cytotoxic topoisomerase II inhibitor which we recently found to undergo a dramatic compartmentalization in lysosomes [25]; hence, in order to exert its topoisomerase II inhibitory activity, C-1330 must reach the nucleus. MCF-7 cells which contain a low number of lysosomes per cell displayed a high confinement of C-1330 fluorescence in their nuclei (Fig. 1). In contrast, C-1330 fluorescence in MDR A549/K1.5 cells which contain a high number of lysosomes was predominantly confined to lysosomes rather than to the nucleus. This finding is an initial evidence for a likely correlation between the elevated number of lysosomes per cell, and the lysosome-dependent protection of intracellular drug target sites from cytotoxic drug activity.

### Increased lysosome number per cell correlates with inherent resistance to sunitinib

**Figure 2:**
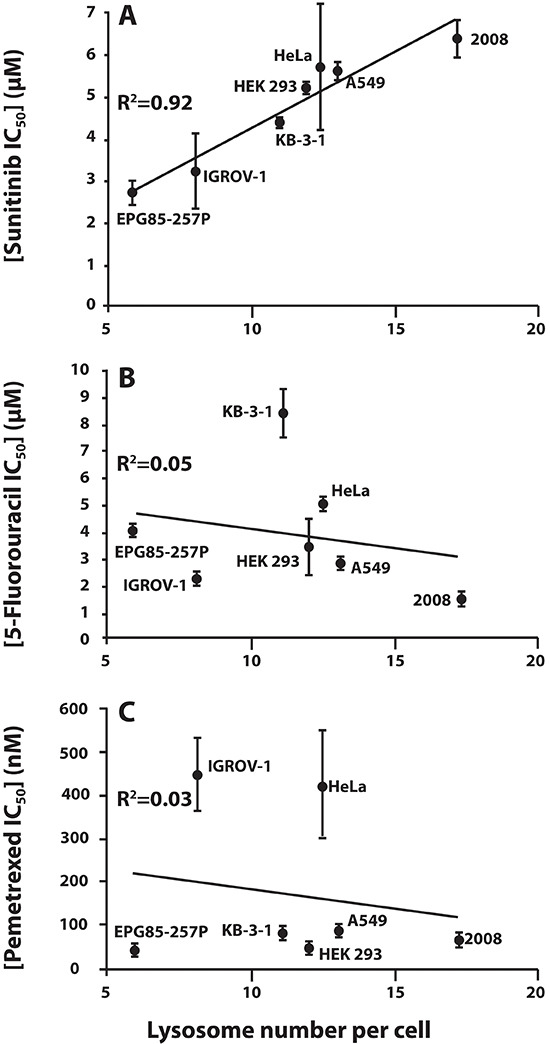
The correlation between lysosome number and intrinsic resistance to the hydrophobic weak base tyrosine kinase inhibitor sunitinib, but not to the hydrophilic thymidylate synthase inhibitors 5-fluorouracil and pemetrexed Seven human carcinoma cell lines of diverse tissue lineage were analyzed for sunitinib **(A)**, 5-fluorouracil **(B)**, and pemetrexed **(C)** cytotoxicity using a colorimetric XTT cell proliferation assay (IC_50_ values are shown on the Y axis), as well as for lysosome number per cell. The latter was performed by monitoring the number of green fluorescent lysosomes (i.e. sunitinib-accumulating lysosomes and acidic vesicles; shown on the X axis) determined using the fluorescence microscope InCell analyzer, preceded by lysosome staining with 10 μM sunitinib (green fluorescence) for 30 min, and computationally analyzed using the InCell investigator software.

Based on these results, we further hypothesized that cells harboring a higher number of lysosomes may exhibit an increased intrinsic resistance to hydrophobic weak base drugs that are prone to undergo marked sequestration in lysosomes. To test this hypothesis, several drug-naïve human tumor cell lines of epithelial origin (i.e. carcinoma) were used including: ovarian carcinoma (2008 and IGROV-1), non-small cell lung cancer (A549), cervical carcinoma (HeLa), nasopharyngeal carcinoma (KB-3-1), gastric carcinoma (EPG85-257P) as well as normal human embryonic kidney cells (HEK293). The different cell lines were tested for their sensitivity to the protein tyrosine kinase inhibitor sunitinib, which we have recently shown to highly accumulate in lysosomes [23]. We also quantified the number of lysosomes per cell in all these tumor cell lines using fluorescence microscopy. We observed a striking linear correlation (R^2^ = 0.92) between mean lysosome number per cell and the inherent resistance of the various tumor cell lines to sunitinib (Fig. 2A). These results suggest that increased accumulation of sunitinib in lysosomes confers upon the tumor cells an intrinsic resistance to this hydrophobic weak base drug. To determine whether this innate resistance is indeed dependent on lysosomal sequestration of the drug, the correlation of the lysosome number in the above tumor cell lines was also compared to the cytotoxic activity of two widely used anticancer drugs that do not undergo lysosomal sequestration, the fluoropyrimidine 5-fluorouracil (5-FU), and the antifolate pemetrexed (PMX), both of which inhibit thymidylate synthase, a key enzyme in pyrimidine nucleotide biosynthesis (Fig. 2B and 2C, respectively). No correlation whatsoever was observed between lysosome number per cell and tumor cell sensitivity to these established anti-tumor agents. These findings suggest that increased lysosome number per cell is likely to serve as a marker for inherent resistance to hydrophobic weak base drugs that are prone to undergo marked accumulation in lysosomes.

### Exposure of MCF-7 cells to hydrophobic weak base drugs induces an increase in lysosome number

**Figure 3:**
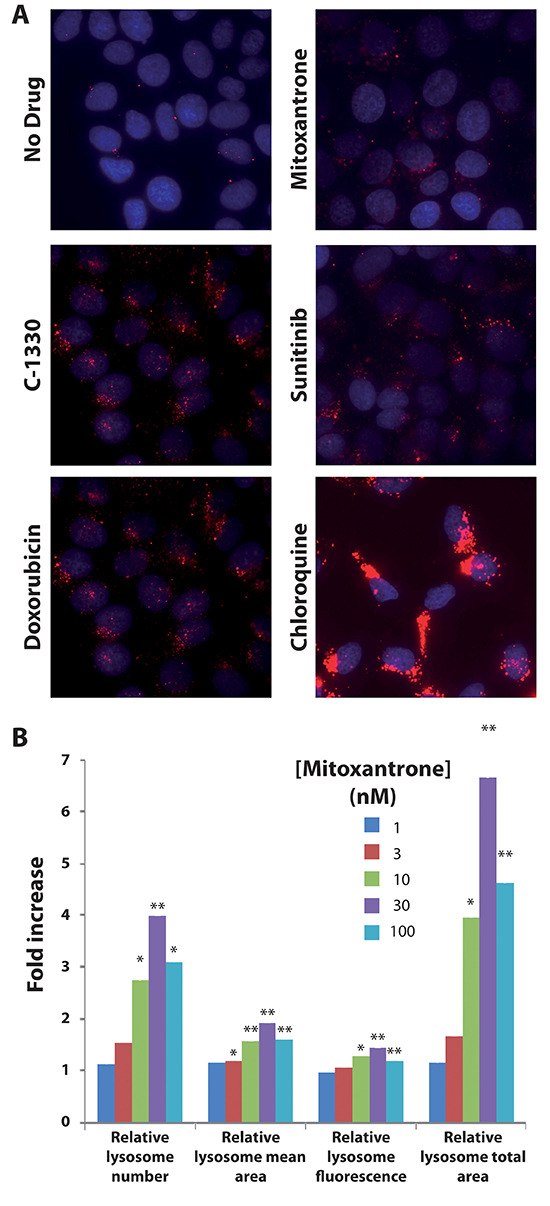
Hydrophobic weak base drugs induce lysosomal biogenesis in MCF-7 breast cancer cells MCF-7 cells were exposed for 72 hr to a single dose of 100 nM mitoxantrone, doxorubicin, sunitinib and C-1330 as well as 100 μM chloroquine. Following drug exposure, cells were stained with 100 nM LysoTracker red for 1 hr and LysoTracker red fluorescence was determined using an InCell analyzer fluorescence microscope **(A)**. We also examined the dose-dependent increase in lysosomal content after exposure of MCF-7 cells to a single dose of mitoxantrone for 72 hr. MCF-7 cells were exposed to increasing concentrations of mitoxantrone (1–100 nM) for 72 hr. Cells were then stained with LysoTracker red and the red fluorescence was determined by fluorescence microscopy. Lysosome number, size and fluorescence intensity were determined using an InCell investigator software **(B)**. Statistical significance is denoted by *(p < 0.05) and **(*p* < 0.01).

Based on previous evidence that lysosomal stress can trigger lysosomal biogenesis [16], we further postulated that lysosomal entrapment of hydrophobic weak base drugs may also result in enhanced lysosomal biogenesis, leading to a further increase in the number of lysosomes per cell. Towards this end, MCF-7 cells which contain a low number of lysosomes per cell, were exposed for 24 hr to a single dose (100 nM) of several cytotoxic drugs that were previously shown to undergo lysosomal sequestration including doxorubicin, mitoxantrone, C-1330 and sunitinib [23–25]. The anti-malarial drug chloroquine (100 μM), a well-established lysosomotropic agent which we recently showed to induce a marked increase in lysosome size and number [25], was used here as positive control. A marked increase in the number of lysosomes per cell was observed for all of these hydrophobic weak base drugs by fluorescence microscopy (Fig. 3A). In order to demonstrate the dose-dependent relationship of the drug-induced increase in lysosome number, MCF-7 cells were exposed to increasing concentrations of mitoxantrone for 72 hr, followed by viable cell staining with LysoTracker red, and were assessed by fluorescence microscopy and computational analysis. The results of this computational analysis that are depicted in Fig. 3B were obtained based on an examination of 40 microscope fields, representing 500–1000 cells from each sample. This analysis revealed a 2.7-fold increase in the number of lysosomes per cell after an exposure to sub-cytotoxic mitoxantrone concentrations as low as 10 nM, representing less than 6% of the IC_50_ (which was 170 nM for a 72 hr mitoxantrone treatment) as determined by a colorimetric XTT cell proliferation assay. The maximal increase in lysosomal number per cell in MCF-7 cells was achieved with 30 nM mitoxantrone, which led to a 3.9-fold increase in the number of lysosomes per cell, along with a 1.9-fold increase in the mean size of lysosomes (determined as the cross-sectional lysosomal area) as well as a mean of 1.4-fold increase in lysosome fluorescence intensity (an indicator of lysosome ability to accumulate LysoTracker red). Collectively, these results indicate an estimated increase of up to 16.4-fold in the total volume of cell lysosomes upon exposure to 30 nM mitoxantrone.

### Drug-induced lysosomal biogenesis is associated with the translocation of TFEB from the cytoplasm to the nucleus in malignant and non-malignant human cell lines

**Figure 4:**
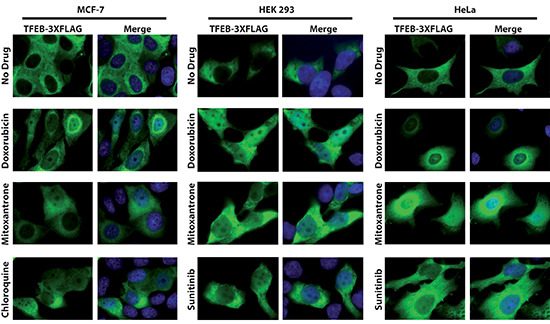
Translocation of TFEB-FLAG from the cytoplasm to the nucleus after exposure of malignant and non-malignant human cells to doxorubicin, mitoxantrone and sunitinib Stably transfected MCF-7 TFEB-3XFLAG cells were exposed to 0.5 μM doxorubicin, 0.5 μM mitoxantrone or 100 μM chloroquine for 24 hr. In an independent set of experiments, 24 hr after transient transfection of HEK293 and HeLa cells with FLAG-tagged TFEB using electroporation, The cells were exposed to 0.5 μM doxorubicin, mitoxantrone, or sunitinib for 24 hr. Cells were then fixed, stained with the DNA dye DAPI (blue fluorescence), incubated with an anti-FLAG antibody (green fluorescence) and analyzed by a fluorescence microscope. The first row represents the drug-free control cells.

**Figure 5:**
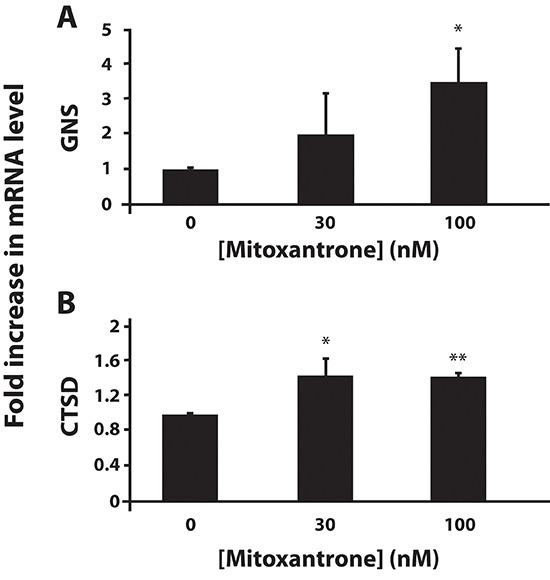
Exposure of MCF-7 cells to mitoxantrone induces an increase in gene expression of the established lysosomal enzyme markers GNS and CTSD MCF-7 cells were exposed to various concentrations of mitoxantrone for 24 hr. Then, GNS **(A)** and CTSD **(B)** gene expression levels were determined using quantitative real time PCR. Statistical significance is denoted by *(*p* < 0.05) and **(*p* < 0.01).

We next explored the factors regulating hydrophobic weak base drug-induced lysosomal biogenesis. In this respect, TFEB, a master transcription regulator, was previously shown to trigger lysosomal biogenesis upon its translocation from the cytoplasm to the nucleus [14]. To examine the impact of hydrophobic weak base drug exposure on the possible translocation of TFEB from the cytoplasm to the nucleus, three human malignant and non-malignant cell lines were transfected with FLAG-tagged TFEB using both stable and transient transfection approaches. MCF-7 cells were stably transfected with FLAG-tagged TFEB, resulting in the stable transfectant MCF-7-TFEB-FLAG; these were exposed to mitoxantrone or doxorubicin for 24 hr, followed by immunofluorescence microscopy using an anti-FLAG antibody. HeLa and HEK 293 cells were transiently transfected with FLAG-tagged TFEB; twenty four hours later, these cells were exposed to sunitinib, mitoxantrone or doxorubicin for 24 hr followed by immunofluorescent staining using an anti-FLAG antibody (Fig. 4). Prior to drug exposure, FLAG-tagged TFEB was predominantly localized in the cytoplasm in all three cell lines studied, hence being in agreement with previous studies [14, 16]. In contrast, upon exposure to mitoxantrone, doxorubicin, sunitinib, or the lysosomotropic agent chloroquine which was used in MCF-7 cells as a positive control, a marked increase in the translocation of FLAG-tagged TFEB from the cytoplasm to the nucleus was observed. Hence, these findings support our hypothesis that exposure to hydrophobic weak base drugs induces translocation of TFEB to the nucleus and consequent lysosomal biogenesis. To rule out the possibility that transfection reagents induce alterations in subcellular localization of TFEB, we employed a different transfection technique using a linear polyethylenimine-based transfection reagent (JetPEI™); the results showed the same translocation of TFEB from the cytoplasm to the nucleus upon drug exposure in all cell lines studied (data not shown). To confirm that this nuclear translocation of TFEB was associated with an actual transactivation of lysosomal gene expression, we performed quantitative real-time PCR analysis to determine the gene expression levels of glucosamine (N-Acetyl)-6-sulfatase (GNS) and cathepsin D (CTSD); GNS and CTSD are *bona fide* lysosomal genes in the CLEAR pathway that were previously shown to be up-regulated upon activation of lysosomal biogenesis as part of the TFEB-regulated CLEAR gene network [16]. As expected from the marked nuclear localization of TFEB upon exposure to lipophilic weak base drugs, the gene expression levels of both GNS (Fig. 5A) and CTSD (Fig. 5B) were significantly elevated after 24 hr exposure of MCF-7 cells to mitoxantrone. These results provide the first direct evidence for hydrophobic weak base chemotherapeutic drug-induced increase in lysosome number in cancer cells, hence indicating that single dose exposure to lipophilic weak base drugs, such as mitoxantrone, triggers enhanced lysosomal biogenesis in cancer cells.

**Figure 6:**
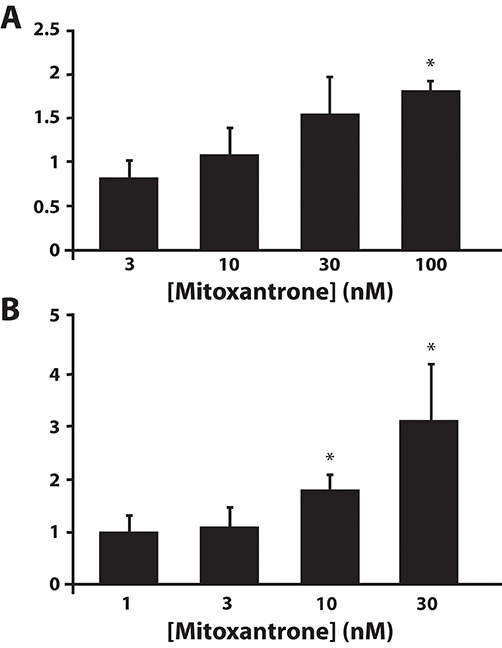
Mitoxantrone induces an increase in lysosomal enzyme activity in a drug dose-dependent manner β-Hexosaminidase activity was determined in MCF-7 cells after their exposure to increasing concentrations of mitoxantrone for 24 hr **(A)** or 72 hr **(B)**. Statistical significance is denoted by *(*p* < 0.05).

We next assessed whether or not the increases in both the mRNA levels of these lysosomal markers as well as in lysosome number per cell were associated with a consistent increase in the catalytic activity of the established lysosomal enzyme β-hexosaminidase. We have previously shown that β-hexosaminidase is a *bona fide* functional marker of the number of lysosomes per cell [29]. MCF-7 cells were exposed to increasing concentrations of mitoxantrone, followed by extraction of cell lysates and determination of β-hexosaminidase activity. A dose-dependent increase in β-hexosaminidase activity was induced by mitoxantrone concentrations as low as 30 nM after 24 hr drug exposure (Fig. 6A). After 72 hr of drug exposure, the increase in β-hexosaminidase activity was evident at drug concentrations as low as 10 nM (Fig. 6B).

**Figure 7:**
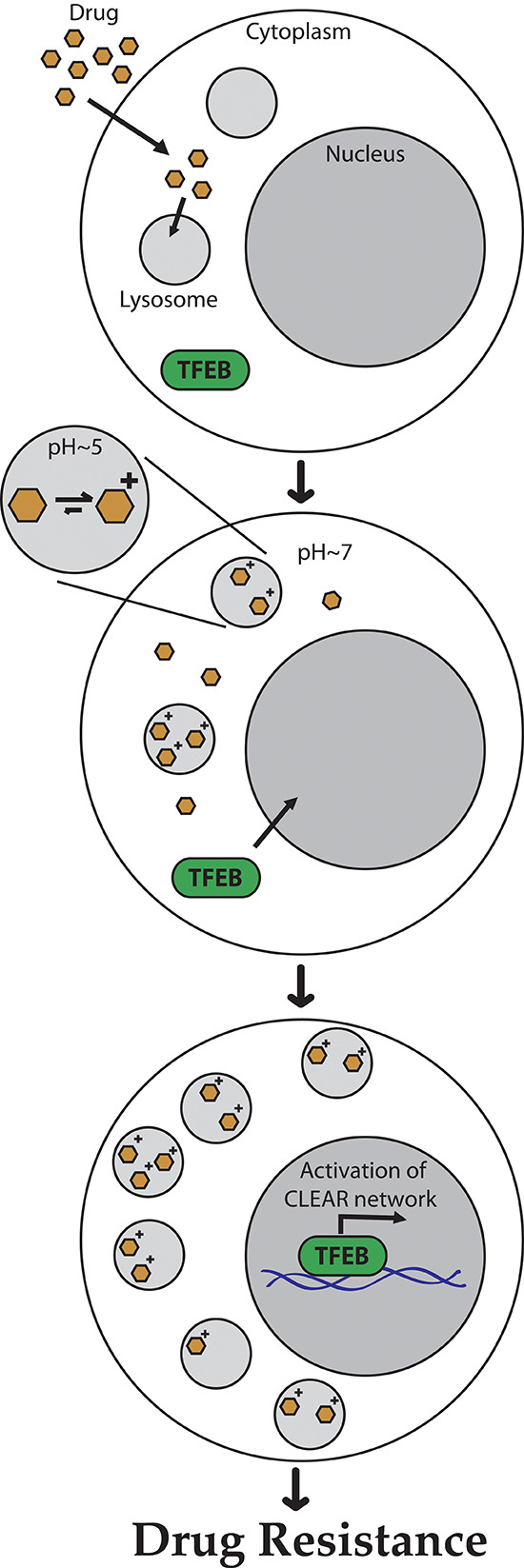
A schematic summary model for hydrophobic weak base drug-induced lysosome-mediated drug resistance Hydrophobic weak base drugs enter the lysosomes by simple diffusion; undergo protonation in the acidic lysosomal lumen, thereby becoming irreversibly sequestered in lysosomes and acidic intracellular vesicles such as late endosomes. In turn, lysosomal drug sequestration triggers TFEB-mediated lysosomal biogenesis, resulting in a significant increase in the number of lysosomes per cell. Increased lysosome number per cell enhances the efficiency of lysosomal drug sequestration, with lysosomes acting as a sink pulling hydrophobic weak base drugs away from their cellular target sites, thereby resulting in MDR.

Based on our current findings we propose an integrative model for hydrophobic weak base drug-induced lysosome-dependent drug resistance (Fig. 7). Hydrophobic weak base drugs enter the lysosomes by simple diffusion, become irreversibly protonated in the highly acidic lysosomes or late endosomes, undergo dramatic accumulation and consequently become irreversibly sequestered therein. In turn, lysosomal drug entrapment triggers TFEB-mediated lysosomal biogenesis via dephosphorylation of TFEB and its translocation from the cytoplasm to the nucleus. In the nucleus, this transcriptional master regulator transactivates the expression of multiple genes in the CLEAR pathway, thereby leading to lysosomal biogenesis and consequently marked increase in lysosome number per cell. Increased lysosomal number per cell increases the efficiency of lysosomal drug sequestration, with lysosomes acting as a sink pulling hydrophobic weak base drugs away from their cellular target sites, hence rendering tumor cells MDR.

## DISCUSSION

Our current findings constitute the first demonstration that the natural variation in lysosome number between various drug-naïve cancer cells is an emerging determinant of the intrinsic resistance to lipophilic weak base cytotoxic agents such as sunitinib. In this respect, we have recently shown that sunitinib undergoes marked sequestration in lysosomes [23]. We therefore propose here that an increased number of lysosomes per cell and consequently enhanced lysosomal entrapment of lipophilic weak base anticancer drugs may prove a novel dismal prognostic factor that correlates with intrinsic resistance to these chemotherapeutics, some of which are listed in Table 1. Accordingly, from a personalized medicine perspective, the number of lysosomes in malignant cells may prove a significant factor that has to be taken into consideration when selecting the treatment of choice for individual cancer patients. Hence, dedicated retrospective studies with various carcinoma specimens exploring the impact of increased number of lysosomes on intrinsic chemoresistance and poor prognosis are warranted.

It has been previously shown that lysosomal stress triggers lysosomal biogenesis [16]. In the current paper several lines of evidence are presented to support our hypothesis that exposure of cancer cells to hydrophobic weak base drugs, and consequent sequestration of these drugs in lysosomes, triggers TFEB-associated lysosomal biogenesis: a) treatment of various drug-naïve cancer cells as well as non-malignant cells with hydrophobic weak base drugs that are highly sequestered in lysosomes, results in a dominant translocation of a FLAG-tagged TFEB from the cytoplasm to the nucleus. These results were consistently obtained both upon transient and stable transfections of FLAG-tagged TFEB, as well as using two distinct transfection techniques including a linear polyethylenimine-based transfection reagent (JetPEI™) as well as electroporation. b) This translocation of TFEB from the cytoplasm to the nucleus resulted in the induction of expression of genes in the CLEAR network including GNS and CTCS. c) This was consistently associated with enhanced lysosomal enzyme activity as evidenced by the dose-dependent increase in the activity of the lysosomal enzyme β-hexosaminidase which we previously used as a lysosomal enzyme marker [29]. These alterations consistently resulted in a marked increase in cell lysosome number, size and capacity, as indicated by the enhanced lysosomal accumulation of LysoTracker red. It is noteworthy that LysoTracker red is sequestered in lysosomes in an acidic pH-dependent manner, much like hydrophobic weak base drugs (Table 1). We therefore propose that the ability of lysosomes to accumulate LysoTracker red in a given cancer cell is a reliable indication of the capacity of these lysosomes to sequester hydrophobic weak base drugs. These findings along with evidence indicating that an elevated lysosome number per cell renders cancer cells resistant to hydrophobic weak base drugs, suggest that lysosome-mediated drug resistance may also be an acquired mechanism of drug resistance, in addition to any pre-existing (i.e. intrinsic) MDR-dependent trait. We therefore propose that targeting the lysosomal biogenesis pathway by circumventing translocation of TFEB from the cytoplasm to the nucleus, may prevent drug-induced lysosome-mediated resistance, thereby increasing the efficacy of anticancer treatment with hydrophobic weak base drugs.

One of the key inevitable questions that arises from the proposed model that we present here (Fig. 7) is how lysosomal accumulation of hydrophobic weak base drugs is perceived by cells as lysosomal damage or stress, hence inducing TFEB-dependent CLEAR pathway, and consequent lysosomal biogenesis. One plausible explanation relies on the established lysosomal alkalinization effect that hydrophobic weak basic compounds have upon marked accumulation in lysosomes. Indeed, exposure to hydrophobic weak base compounds was found to induce lysosomal alkalinization [20, 34, 35], which in turn was found to disrupt lysosomal activity [36]. It is interesting to note that according to this scenario, and given that drug-induced lysosomal biogenesis is mediated by drug-induced lysosomal alkalinization, the basic properties of lysosome accumulating drugs are important not only for the lysosomal sequestration itself, but also for alkalinization of the lysosomal pH, which activates lysosomal biogenesis [14]. Another possible underlying mechanism to constitute this lysosomal insult is presumably based on the fact that a multitude of hydrophobic weak-base drugs contain a dominant hydrophobic polyaromatic ring structure that can facilitate their rapid diffusion into cells and their dramatic intercalation into the lipid core of biomembranes including the lysosomal membrane. Following this favorable co-partition into the lipid-phase of the lysosomal membrane, these amphiphilic amine compounds undergo a dominant protonation in the highly acidic lumen (i.e. pH 5.2) of the lysosomes [37]. As such, this positively charged amine(s) cannot reside in the lipid bilayer of the lysosomal membrane and hence must protrude into the hydrophilic lumen of the lysosomes. Thus, since the typical log *P* value of these hydrophobic weak base compounds can, in some cases, reach values higher than 4 (Table 1), such a drug that is present at an extracellular concentration of only 1 μM which is clinically relevant, will favorably co-partition into the lipid phase of the lysosomal membrane and reach a concentration of 10 mM while its protonated amine(s) protrudes into the hydrophilic acidic lumen of the lysosomes. According to this scenario, these hydrophobic weak base drugs may act as amphipathic detergent-like compounds which destabilize the lysosomal membrane and render it leaky, a phenomenon known as lysosomal membrane permeabilization [38], thereby triggering lysosomal biogenesis. In fact, this proposed model of lysosomal membrane leakage by hydrophobic weak base drugs has been recently demonstrated for another hydrophobic weak base compound siramesine, in cancer cells both *in vitro* and *in vivo* [39–41]. Specifically, siramesine, a novel sigma-2 receptor agonist, induced caspase-independent programmed cell death in immortalized and transformed cells of various origins. Moreover, siramesine-treated tumor cells displayed lysosomal membrane permeabilization, increased levels of reactive oxygen species, chromatin condensation, as well as shrinkage and detachment of tumor cells. Moreover, administration of well-tolerated siramesine doses achieved a marked antitumor activity in mice harboring orthotopic breast cancer and sub-cutaneous fibrosarcoma tumors. It was further shown that siramesine acts as a lysosomotropic detergent which induces a rapid rise in lysosomal pH that is followed by lysosomal leakage and dysfunction [40]. Recently, mechanism-based fluorescence spectroscopy studies identified two distinct binding sites for siramesine in phospholipid membranes with the highest binding affinity, measured for phosphatidic acid [42]. Hence, upon protonation, this amphiphilic amine compound which is presumably well-embedded via its lipid-soluble aromatic ring structure in the lipid core of the lysosomal membrane, ionically interacts with anionic phospholipids hence resulting in lysosomal membrane permeabilization and leakage [38]. Taking advantage of this lysosomal membrane permeabilization, a recent study showed the remarkable antitumor activity *in vitro* and *in vivo* of the combination of siramesine and vincristine, the latter of which is a microtubule-destabilizing agent known to induce dramatic lysosomal alterations, thereby offering a novel combination cancer therapy [41].

From a cancer therapy perspective, it has been suggested that lysosomes can serve as a novel emerging target for anticancer treatment [43] via various approaches of lysosomal disruption by lysosomotropic agents such as mefloquine [44], lysosomal photodestruction by lysosome accumulating photosensitizes such as imidazoacridinones [25], or induction of lysosome-mediated apoptosis [45]. Based on the data presented herein we postulate that cells with a higher lysosome number are likely to be more resistant to many hydrophobic weak base chemotherapeutics currently used including mitoxantrone, doxorubicin, sunitinib and other compounds listed in Table 1. In the same time, tumor cells which are rich in lysosomes may exhibit a favorable response to treatment that targets lysosomes. Thus, targeting lysosomes in malignant cells may prove a viable and efficacious strategy to eradicate drug resistant tumor cells. This hypothesis is strongly supported by recent studies which targeted lysosomes in cancer cells both *in vitro* and *in vivo*. The group of Jaattella recently showed that lysosomal membrane permeabilization and subsequent cell death may prove useful in cancer treatment, provided that cancer cell lysosomes can be selectively targeted [46]. In a different study, we have recently reported that certain imidazoacridinones highly accumulate in lysosomes and can be used as photosensitizers to induce an imidazoacridinone-dependent lysosomal photodestruction, as exposure to light resulted in rapid photo-rupture of imidazoacridinone-loaded lysosomes and cell death [25]. In this respect, we have shown that lysosomal photodestruction of MDR cells reversed the MDR phenotype, thereby restoring parental cell sensitivity. Hence, these findings suggest that lysosome-mediated photodestruction can be used as a pharmacological ‘Trojan horse’ to overcome drug resistance in cancer cells.

## MATERIALS AND METHODS

### Chemicals

LysoTracker red DND99 was purchased from Invitrogen (Carlsbad, CA, USA). 4-Nitrophenyl N-acetyl-β-D-glucosaminide, 4′,6′-diamidino-2-phenylindole (DAPI), chloroquine and mitoxantrone were from Sigma-Aldrich (St. Louis, MO, USA). Doxorubicin was from Tocris Bioscience (Bristol, United Kingdom). Sunitinib was a kind gift from Prof. A.W. Griffioen, VU Medical Center, Amsterdam, The Netherlands. The imidazoacridinone C1330 was kindly provided by Prof. A. Skladanowski, Gdansk University of Technology, Gdansk, Poland.

### Cell culture and transfections

MCF-7 breast cancer, 2008 and IGROV-1 ovarian carcinoma, A549 and A549/K1.5 non-small cell lung cancer, HeLa cervical carcinoma, KB-3-1 nasopharyngeal carcinoma, EPG85-257P gastric carcinoma and HEK293 human embryonic kidney cells used in the current study, were maintained in RPMI-1640 medium (Gibco, Paisley, UK), supplemented with 10% fetal bovine serum, 2mM glutamine, 100 μg/ml penicillin and streptomycin (Biological Industries, Beth-Haemek, Israel) in a humid atmosphere containing 5% CO_2_ at 37°C. MCF-7, HeLa and HEK293 cells were transfected by electroporation (1000 μF, 234 V) with an expression plasmid (10 μg plasmid DNA; kindly provided by Prof. A. Ballabio, Telethon Institute of Genetics and Medicine, Naples, Italy) containing FLAG-tagged TFEB (3XFLAG-TFEB) using 4 mm electroporation cuvettes from Cell Projects (Kent, UK) and the electroporation system Gene Pulser II (Bio-Rad, Hercules, CA). To obtain stably FLAG-TFEB transfected cells, MCF-7 cells were transfected and 24 hr after transfection they were subjected to G-418 selection (500 μg/ml; Sigma-Aldrich, St. Louis, MO, USA) in the growth medium. HEK293 and HeLa cells were also transiently transfected with the expression plasmid containing FLAG-tagged TFEB using the JetPEI transfection reagent (Polyplus-transfection; Genesee Scientific, San Diego, CA) according to the instructions of the manufacturer.

### Live cell imaging and quantification of lysosomes

MCF-7 cells were plated in 24-well glass bottom plates (*In Vitro* Scientific, CA, USA). For subcellular drug localization studies, live cells were incubated with 10 μM C-1330 for 30 min along with 100 nM LysoTracker red for 1 hr at 37°C. Fluorescence of C-1330 and the lysosomal marker LysoTracker red was followed with Zeiss inverted Cell-Observer microscope. To determine the impact of the anticancer drug on lysosomes, MCF-7 cells were incubated for 24 hr in growth medium containing100 nM mitoxantrone, doxorubicin, sunitinib or C-1330, or 100 μM chloroquine, and viably co-stained with 100 nM LysoTracker red for 1 hr. To achieve nuclear staining prior to fluorescence imaging, cells were incubated with 2 μg/ml Hoechst 33342 in growth medium for 10 min. LysoTracker red fluorescence was followed with a fluorescence microscope InCell analyzer 2000 (GE Healthcare Bio-Sciences, Pittsburgh, PA, USA). Computational analysis of lysosome number, lysosome size and LysoTracker red fluorescence intensity was determined using InCell investigator software.

### Immunofluorescence microscopy studies

Stable transfectants of MCF-7 cells expressing FLAG-tagged TFEB were seeded in 24-well plates on sterile glass coverslips and incubated for 24 hr at 37°C. Cells were then incubated with 0.5 μM mitoxantrone, 0.5 μM doxorubicin or 100 μM chloroquine for 24 hr. Cells were then fixed with 4% formaldehyde for 30 min and permeabilized with 0.1% Triton X-100 in phosphate-buffered saline (PBS) at pH 7.4 for 5 min. FLAG-tagged TFEB was visualized using mouse monoclonal anti-FLAG antibody (1:1000 dilution, Sigma-Aldrich St. Louis, MO, USA). During the incubation with the secondary antibody, cell nuclei were counterstained with the DNA dye DAPI (0.5 μg/ml). Coverslips were mounted onto glass slides using Fluoromount-G (Southern Biotechnology Associates, Birmingham, AL) and examined using Zeiss inverted Cell-Observer microscope. Merged images were obtained using the AxioVision program (Zeiss).

### RNA extraction and quantification of lysosomal gene expression by real-time PCR

MCF-7 cells were seeded in 6-well plates and incubated for 24 hr at 37°C. Cells were then incubated with increasing concentrations of mitoxantrone for 24 hr or 72 hr. RNA extraction and cDNA synthesis were carried out as previously described [47]. Gene expression levels of glucosamine (N-Acetyl)-6-Sulfatase (GNS) and cathepsin D (CTSD) were determined using a quantitative real-time PCR assay as previously described [47]. Gene expression levels were normalized using the β_2_-microglobulin (B_2_M) gene as an internal control.

### β-hexosaminidase activity assay

MCF-7 cells were seeded in 6-well plates and incubated for 24 hr at 37°C. Cells were exposed to increasing concentrations of mitoxantrone for 24 hr or 72 hr. Cells were then lysed using a Potter-Elvehjem PTFE Tissue Grinder (Kontes Glass Co, Vineland, NJ, USA). β-hexosaminidase activity in cell lysates was assayed using 4-Nitrophenyl N-acetyl-β-D-glucosaminide as a chromogenic substrate as previously described [29].

### Cytotoxicity assay

Cells were seeded in 96-well plates and incubated overnight at 37°C. Cells were then exposed to increasing concentrations of sunitinib for 72 hr. Cytotoxicity analysis was undertaken using a colorimetric XTT cell proliferation assay (Biological Industries, Beth-Haemek, Israel) according to the instructions of the manufacturer.

### Statistical analysis

Results presented in the current study were obtained from three or more independent experiments performed on separate days. Error bars in all graphs and charts represent standard deviation. The statistical significance of a difference was evaluated using the unpaired two-sided Student's t-test.
