# Correction: Lysosomal sequestration of hydrophobic weak base chemotherapeutics triggers lysosomal biogenesis and lysosome-dependent cancer multidrug resistance

**DOI:** 10.18632/oncotarget.28110

**Published:** 2022-04-01

**Authors:** Benny Zhitomirsky, Yehuda G. Assaraf

**Affiliations:** ^1^The Fred Wyszkowski Cancer Research Laboratory, Dept. of Biology, Technion-Israel Institute of Technology, Haifa 32000, Israel

**This article has been corrected:** In Figure 3A, the ‘C-1330’ panel is an accidental duplicate of the ‘Doxorubicin’ panel. The corrected Figure 3, produced using the original data, is shown below. The authors declare that these corrections do not change the results or conclusions of this paper.

Original article: Oncotarget. 2015; 6:1143–1156. 1143-1156. https://doi.org/10.18632/oncotarget.2732

**Figure 3:**
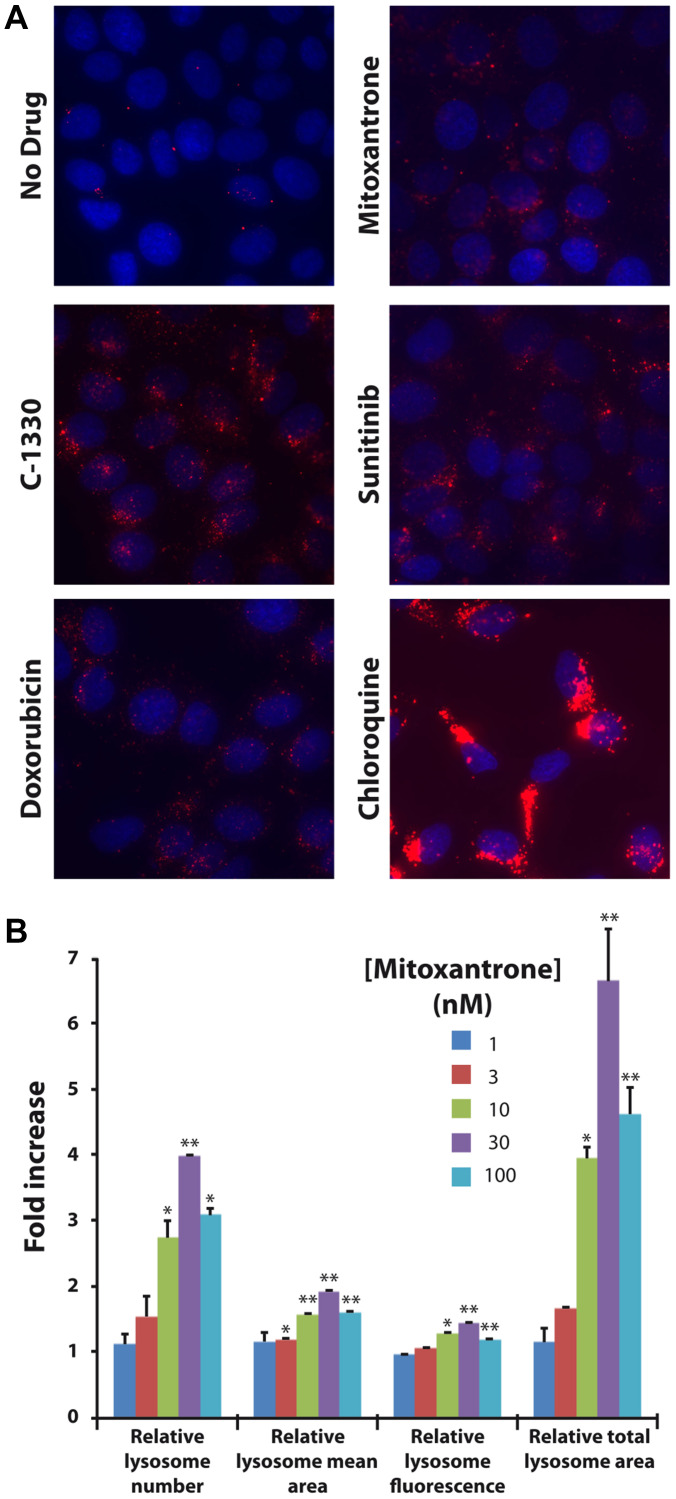
Hydrophobic weak base drugs induce lysosomal biogenesis in MCF-7 breast cancer cells. MCF-7 cells were exposed for 72 hr to a single dose of 100 nM mitoxantrone, doxorubicin, sunitinib and C-1330 as well as 100 μM chloroquine. Following drug exposure, cells were stained with 100 nM LysoTracker red for 1 hr and LysoTracker red fluorescence was determined using an InCell analyzer fluorescence microscope (**A**). We also examined the dose-dependent increase in lysosomal content after exposure of MCF-7 cells to a single dose of mitoxantrone for 72 hr. MCF-7 cells were exposed to increasing concentrations of mitoxantrone (1–100 nM) for 72 hr. Cells were then stained with LysoTracker red and the red fluorescence was determined by fluorescence microscopy. Lysosome number, size and fluorescence intensity were determined using an InCell investigator software (**B**). Statistical significance is denoted by (^*^ *p* < 0.05) and (^**^ *p* < 0.01).

